# Simplifying research access to genomics and health data with Library Cards

**DOI:** 10.1038/sdata.2018.39

**Published:** 2018-03-14

**Authors:** Moran N. Cabili, Knox Carey, Stephanie O. M. Dyke, Anthony J. Brookes, Marc Fiume, Francis Jeanson, Giselle Kerry, Alex Lash, Heidi Sofia, Dylan Spalding, Anne-Marie Tasse, Susheel Varma, Ravi Pandya

**Affiliations:** 1 Broad Institute of MIT and Harvard, Cambridge, MA 02142, USA; 2 Mountain View, CA 94043, USA; 3 McGill University, Montreal, Canada H3A 0G1; 4 University of Leicester, Department of Genetics & Genome Biology, Leicester, LE1 7RH, UK; 5 DNAstack, Toronto, Canada M5G 1M1; 6 IAMOPEN, Toronto, Canada M4E 2J1; 7 European Molecular Biology Laboratory (EMBL-EBI), Cambridge, CB10 1SD, UK; 8 Simons Foundation, New York, NY 10010, USA; 9 NHGRI, NIH, Bethesda, MD 20892, USA; 10 Microsoft, Redmond, WA 98052, USA

**Keywords:** Research data, Medical research

## Abstract

The volume of genomics and health data is growing rapidly, driven by sequencing for both research and clinical use. However, under current practices, the data is fragmented into many distinct datasets, and researchers must go through a separate application process for each dataset. This is time-consuming both for the researchers and the data stewards, and it reduces the velocity of research and new discoveries that could improve human health. We propose to simplify this process, by introducing a standard Library Card that identifies and authenticates researchers across all participating datasets. Each researcher would only need to apply once to establish their bona fides as a qualified researcher, and could then use the Library Card to access a wide range of datasets that use a compatible data access policy and authentication protocol.

## Introduction

The global research community is collecting ever-increasing volumes of genomics & health data that could be used to advance our understanding and treatment of human health. However, it is difficult to extract all the potential benefit from this data, because it is divided into thousands of individual datasets, and each researcher must separately apply for access to each dataset through a distinct Data Access Committee (DAC). These DACs exist for an important purpose, to ensure that researchers abide by the terms under which the data was collected, and to safeguard privacy of the human research subjects. For example, a dataset might only be available for non-commercial research related to cancer. However, the DAC approval process is time-consuming for researchers, and discourages them from using all the available data relevant to their research. Furthermore, it results in duplication of efforts as each DAC needs to separately verify the identity of a researcher along with their credentials and institutional affiliations, and currently there is no mechanism which leverages the work one DAC completed as a reference for another DAC. Moreover, the requirements for identity verification are not standardized across DACs.

The Global Alliance for Genomics and Health (GA4GH) is an international organization with the mission to enable effective and responsible sharing of genomic and clinical data^1^. The GA4GH Library Card project is working to make the process of identity verification simpler and faster, for both researchers and DACs, while still satisfying the compliance and privacy goals of the DACs. A researcher would only need to go through a single validation process to verify their identity and credentials, and could then use their Library Card with any compatible dataset. A DAC would only need to specify their requirements for identity validation and researcher credentials using a standard vocabulary, and would no longer need to manually verify each individual.

The Library Card would also support new developments in data access, such as the GA4GH Registered Access model^2^. Registered Access aims to provide access to users within defined ‘roles’ (e.g., researcher, clinician) to integrated data resources such as those planned for the Beacon Project (http://beacon-network.org). Access would therefore hinge on the identity and role of users, as well as on their agreement to a general set of data use conditions, but would not be tied to a specific project or research proposal.

As the volume of genomic data grows, and moves to data repositories hosted in public clouds where researchers can also run computations on-demand^3^, the standard identity and authentication mechanism provided by the Library Card will be essential for simplifying access to data. These new models for hosting and analyzing genomic data reduce the barrier to entry and make analysis on genomic data accessible to a wider range of researchers, including those without internal resources to perform storage and compute on big data. Development of efficient mechanisms for data use oversight, such as use of a Library Card to verify researcher’s identity, are complementary efforts for making compliant data analysis accessible to more researchers.

## Library Cards

The Library Cards group within GA4GH is working on an initial prototype, starting with a limited set of use cases, such as academic researchers accessing Registered Access datasets. We will extend the model over time to cover more general use cases.

The Library Card model consists of four elements:

1. A standard real-world process for verifying a researcher’s identity, and relating it to an online identity
2. A standard model for electronically representing a researcher’s identity and access-related information
3. A standard model for a DAC to electronically represent their policy for who is allowed to access a dataset
4. A standard protocol for servers to authenticate and authorize a user who is requesting access to a dataset

We envision the experience of using a Library Card as similar to the common web experience of using a ‘Sign in with Facebook’ button instead of creating a unique account for each website, and based upon the same open, interoperable technology standards. The user might start at a web portal for a genomic data resource, such as a dataset for a genomic study of diabetes deposited in the NIH’s Database of Genotypes and Phenotypes (dbGaP). There they would see a ‘Sign in with Library Card’ button, which would take them to the Library Card website. If they do not yet have a Library Card, they would need to create one.

In order to acquire a library card, users will need to provide some information validating both their physical identity and their researcher status. Users will also need to agree to abide by a set of principles to maintain the security of the data, to avoid attempting to re-identify subjects, etc, and complete an online training session.

The system will then generate for them an electronic object which represents their Library Card, containing a standard set of claims about their identity, the credentials they provided, and the process used to validate their identity. A dataset owner can then check the identity & claims in the Library Card before granting them access to the data.

In the future, if they are also applying for controlled access data, then there might be some additional information required, such as research purpose (e.g. cancer, neurological diseases, methods development), geographical location, etc. This will be translated into attributes that can be evaluated against dataset requirements using Consent Codes, DUOS, or ADA-M (as described below).

We envision that the Library Card system will be comprised from a federated network of recognized entities. Some of these entities will be entitled to issue Library Cards, while others would verify claims on the card, while all complying to the same standard protocol. We hope to leverage and bridge existing initiatives with similar goals such as ORCID (http://orcid.org) and the NIH eRA Commons.

We are currently at the early stages of designing the technical and practical solution for growing the Library Card system. It will be important to ensure that the identity verification process is simple for the researcher, but also sufficient to satisfy the requirements of the data provider. The existing model for controlled access data ultimately relies on an individual from the institution agreeing to follow a set of information security guidelines, including access control and authentication for the researchers. For example, the European Genome-phenome Archive (EGA) (https://ega-archive.org) DACs require the researcher to sign a data access agreement between the DAC and their institution; the NIH eRA Commons requires a signing official to agree to such policies as part of each dbGaP access request. EGA and dbGaP each have several thousand researchers registered under this process.

We plan to build on this existing infrastructure, for example by using an initial enrollment step when an institution joins the Registered Access system, and asking the signing official to certify that their identity management practices meet a standard set of requirements. This certification provides a basic level of assurance so that Library Cards can use institutional identity for authenticating researchers. This can be extended over time to other groups, such as clinical care professionals or citizen scientists; they could establish their researcher bona fides using professional credentials, publication records, or attestation by another researcher, and verify their identity through real-world identity validation services such as Experian Inc.

## Authorization and authentication protocols

The integrity of the Library Cards system depends upon proper identity proofing, authentication, and authorization. To promote ease of integration and maximum interoperability, the authentication and authorization subsystems will be based upon widely-adopted open standards such as OAUTH 2.0 (https://tools.ietf.org/html/rfc6749) and OpenID Connect (http://openid.net/developers/specs/). These technologies allow Library Card issuers to act as trusted identity providers to multiple data holders, freeing them from the day-to-day burdens of managing email addresses, two-factor authentication codes, lost passwords, and so forth. Historically, authentication systems have implemented these features in slightly different, incompatible ways, imposing a tremendous burden on researchers — especially when the research involves data sets across multiple institutions. The DACs, under whose authority data are being provided, would be able to trust that the Library Card issuer has performed the appropriate degree of identity proofing, and is issuing interoperable authentication credentials with consistent semantics.

A Library Card would encode identity and other attributes of a researcher as a set of standardized *claims*. For example, institutional affiliation may be recorded in a claim, along with a level of assurance regarding the claim: (a) self-assertion (b) institutional email (c) proof of support of the claim by an authorized third party within the institution. These claims, and others like them, are recorded by the Library Card issuer and provided over secure protocols to relying parties who will in turn make access decisions based upon these claims. Authorization decisions may also depend upon aspects of the authentication methods used — for example, did the researcher authenticate with a verified email and password, or was a time-based second factor used as well? The standards chosen as the basis for the Library Card authentication and authorization subsystem provide the flexibility to encode these and other claims in a standard way, allowing the community to experiment with different claims structures to achieve the broadest adoption across DACs. These standards include provisions for federating identity, authentication, and claims among a set of cooperating institutions, allowing these credentials to have global usability. We plan to harmonize this model with the emerging W3C Verifiable Claims initiative, to ensure that the Library Card system is as interoperable and standards-based as possible.

## Registered access

The Registered Access model is a policy framework for making genomics and health data available based upon the requester’s role, rather than on a per-project basis. The model defines a set of roles, how these would be established by individuals for the purpose of data access, as well as the attestation required to establish a user’s bona fides and appropriate data use for each role. These are simple and generally applicable criteria: for example, a researcher may acquire Registered Access status by having their home institution verify the claims on their Library Card with regard to the researcher’s affiliation. Alternatively, it may be possible for a researcher whose bona fides have already been established to corroborate claims on the Library Card of a new researcher that is requesting registered access. The standard Registered Access data use restrictions require the user to comply with standard ethical and legal restrictions, and follow the GA4GH Framework for Responsible Sharing of Genomic and Health-Related Data. Our goal is to encourage a growing body of data to be made available under this model and for the Library Card to become the access credential for Registered Access.

## Computable policy for controlled access

There will likely continue to be a large volume of data that cannot be available through the Registered Access model, and thus will fall under a controlled access model. There is still opportunity to simplify and automate some of the controlled access process, and the Library Cards system can play an important role in this. Automated services to facilitate data use oversight must, first, validate each user as legitimate, and, second, authorize the specific research request based on its compatibility with restrictions on the data set originating from patient consents. The Library Card system provides a standard, interoperable framework for the first step, ensuring that the user is attested and authenticated in a role that enables them to access the data. The Library Card protocol could also incorporate the claims required to use controlled access data, so that it can provide a single unified identity experience to the researcher.

For the second step, to electronically authorize research requests to controlled access data, the *data use restrictions* on every dataset in the data repository must be indexed using a *data use ontology*. Researchers then request to access the data using a templated *data access request* that is then mapped to the same ontology. Once these components are standardized to a common language, the task of reviewing and granting access commonly practiced by the Data Access Committee can be simplified, and eventually automated.

The GA4GH team investigating these issues learned that the existing ontologies focus on capturing consent for different types of care and related conditions, but do not capture restrictions on researching the data, such as restricting use to non-commercial, pediatric, or neurological applications. Members of the team therefore developed complementary ontologies and supporting systems, such as Consent Codes^4^, the Data Use Ontology (DUO) (http://www.obofoundry.org/ontology/duo.html), Data Use Oversight System (DUOS) (https://duos.broadinstitute.org), and Automatable Discovery and Access Matrix (ADA-M) (https://www.ga4gh.org/ga4ghtoolkit/regulatoryandethics/), to represent both data access requests and data use restriction in a machine readable form.

The Broad Institute of MIT and Harvard is formally evaluating a pilot trial of DUOS and Consent Codes overseen by Partners’ Healthcare IRB. During this pilot, a DAC comprised of experts in data use oversight (from the NIH, Partners’ IRB, Sage Bionetworks and Broad Institute) evaluate data access requests in parallel with the DUOS software. The initial DUOS system will authenticate users with NIH eRA Commons, which will issue a claim about the user’s role. The DUOS development team plans to implement the Library Card protocols as they emerge, as a robust federated mechanism to establish the identity and *bona fides* of researchers from many institutions.

The GA4GH ADA-M task team was formed in 2015, and recently released version 1.0 of a specification and API to describe atomic conditions of use for biomedical resources, enabling data stewards or access committees to create precise and highly customizable access profiles for their health and genomic data. The ADA-M model can be incorporated into a Data Use Ontology and the Library Cards identity framework.

There have been a number of earlier initiatives to standardize and electronically capture patient consent. Health Level Seven (HL7) is a comprehensive suite of standards for electronic management of healthcare data. It includes the HL7 Privacy and Security Classification System (HCS) standard to record electronically obtained patient consent (https://www.hl7.org/fhir/consent.html, http://www.hl7.org/implement/standards/product_brief.cfm?product_id=345), which has been adopted by many services^5^(https://www.gov.uk/government/publications/review-of-data-security-consent-and-opt-outs). The GA4GH Consent Codes have recently been incorporated into the HL7 Purpose of Use code system for research uses of data. Also, an ontology similar to DUO was developed by UCSD as part of the iDASH project^5^.

EGA is building and deploying systems that are actively working towards these goals. EGA is working with ELIXIR (https://www.elixir-europe.org) to use the ELIXIR AAI (https://www.elixir-europe.org/services/compute/aai), which allows a single identity (based upon OAUTH2 and OpenID Connect) to be shared with EGA and other ELIXIR services. EGA has also been working with GA4GH to develop the Data Use Ontology (DUO), and is starting to apply the DUO to both new and existing datasets submitted to EGA. EGA also has a registered access Beacon, which allows users with an EGA account to access datasets as a registered user without requiring review by a DAC. By linking the ELIXIR AAI, DUO and registered level datasets, EGA aims to support the core technologies which will allow GA4GH Library Cards to facilitate data access to controlled and registered level datasets.

## Discussion

The Library Cards framework aims to provide a robust, distributed, standards-based approach to validating and authenticating users as *bona fide* researchers. This is a key enabling technology to allow researchers to use other GA4GH standards for accessing genomic data and running bioinformatics computations. These standards are being developed in conjunction with a set of Driver Projects that will apply the technologies in a real-world setting, ensuring that they will be useful to the world-wide genomics community. Large-scale projects such as the EGA and the US AllOfUs^TM^ precision medicine initiative are helping shape the standard so that it will evolve into a robust platform that is used widely in the genomics and health community.

We would also welcome collaboration with other fields outside of genomics and health - it would be ideal if we could work towards a common model for researcher identity and authentication across a range of disciplines, to simplify access to data for many areas of scientific discovery.

## Additional information

**How to cite this article**: Cabili, M. N. *et al.* Simplifying research access to genomics and health data with Library Cards. *Sci. Data* 5:180039 doi: 10.1038/sdata.2018.39 (2018).

**Publisher**’**s note**: Springer Nature remains neutral with regard to jurisdictional claims in published maps and institutional affiliations.

## References

[b1] Global Alliance for Genomics and Health. A federated ecosystem for sharing genomic, clinical data. *Science* **352**, 1278–1280 (2016). 10.1126/science.aaf6162 27284183

[b2] Dyke S. O. M. *et al.* Registered access: a ‘Triple-A’ approach. European Journal of Human Genetics 24, 1676–1680 (2016). 27677416 10.1038/ejhg.2016.115 PMC5117919

[b3] Paten B. *et al.* A Data Biosphere for Biomedical Research. *Medium* https://medium.com/@benedictpaten/a-data-biosphere-for-biomedical-research-d212bbfae95d (2017).

[b4] Dyke S. O. M. *et al.* Consent Codes: Upholding Standard Data Use Conditions. PLoS Genet. 12, e1005772 (2016). 26796797 10.1371/journal.pgen.1005772 PMC4721915

[b5] Ohno-Machado L. *et al.* iDASH: integrating data for analysis, anonymization, and sharing. JAMIA 19, 196–201 (2011). 22081224 10.1136/amiajnl-2011-000538 PMC3277627

